# AraNet v2: an improved database of co-functional gene networks for the study of *Arabidopsis thaliana* and 27 other nonmodel plant species

**DOI:** 10.1093/nar/gku1053

**Published:** 2014-10-29

**Authors:** Tak Lee, Sunmo Yang, Eiru Kim, Younhee Ko, Sohyun Hwang, Junha Shin, Jung Eun Shim, Hongseok Shim, Hyojin Kim, Chanyoung Kim, Insuk Lee

**Affiliations:** 1 Department of Biotechnology, College of Life Science and Biotechnology, Yonsei University, Seoul, Korea; 2 Center for Systems and Synthetic Biology, Institute for Cellular and Molecular Biology, University of Texas at Austin, TX 78712, USA

## Abstract

*Arabidopsis thaliana* is a reference plant that has been studied intensively for several decades. Recent advances in high-throughput experimental technology have enabled the generation of an unprecedented amount of data from *A. thaliana*, which has facilitated data-driven approaches to unravel the genetic organization of plant phenotypes. We previously published a description of a genome-scale functional gene network for *A. thaliana*, AraNet, which was constructed by integrating multiple co-functional gene networks inferred from diverse data types, and we demonstrated the predictive power of this network for complex phenotypes. More recently, we have observed significant growth in the availability of omics data for *A. thaliana* as well as improvements in data analysis methods that we anticipate will further enhance the integrated database of co-functional networks. Here, we present an updated co-functional gene network for *A. thaliana*, AraNet v2 (available at http://www.inetbio.org/aranet), which covers approximately 84% of the coding genome. We demonstrate significant improvements in both genome coverage and accuracy. To enhance the usability of the network, we implemented an AraNet v2 web server, which generates functional predictions for *A. thaliana* and 27 nonmodel plant species using an orthology-based projection of nonmodel plant genes on the *A. thaliana* gene network.

## INTRODUCTION

Due to the exponential growth of the population, the human race continues to have an unmet demand for food and energy. To address these problems, plant scientists have been searching for genes that modulate desirable crop phenotypes, such as higher yield and increased resistance to biotic and abiotic stresses. *Arabidopsis thaliana* is a model plant organism that has been widely used to hunt for such genes. Despite several decades of *Arabidopsis* research, our knowledge of gene functions for *Arabidopsis* and nonmodel crop species is still largely limited. For example, we currently have some functional annotations with experimental evidence for approximately 40% of Arabidopsis and 1% of rice protein coding genes (1). Over the past decade, *A. thaliana* researchers have produced significant volumes of genome-scale data using DNA microarray and other technologies. The recent advent of next-generation sequencing (NGS) technology has been expanding the benefit of genomics to many nonmodel plants as well. Indeed, NGS has already resulted in the deposit of an unprecedented volume of sequence data to public databases and has identified hundreds of genomic loci that may be associated with crop phenotypes of agricultural importance. The rapid increase in available data, however, poses a challenge to the existing data analysis methods available to the field of omics-driven crop science.

Networks are one promising approach to integrate and interpret the massive and heterogeneous genomic data. A network of molecular interactions mined from genomics data may reconstruct a holistic functional organization of cellular or organismal systems. Using various graph algorithms, we may be able to predict not only gene-to-function but also gene-to-phenotype associations (2). We previously constructed a genome-scale functional network for *A. thaliana*, AraNet, and demonstrated its effectiveness in identifying novel genes for important plant phenotypes such as root growth and drought resistance (3). Since the initial development of AraNet, a large volume of new microarray data derived from more diverse biological contexts has been added to the publicly available databases (4). In addition, several genome-scale protein–protein interaction data sets have been published (5–8). We have also continued to improve machine learning algorithms for inferring functional gene associations from genomics data. At this time, therefore, we are in a unique position to introduce an updated database of co-functional networks for *A. thaliana*.

In this paper, we present AraNet v2, which includes new data sets and new computational methods. We demonstrate significant improvements in the predictive power of AraNet v2 using a validation data set that is independent from the data used for network construction. The network web server also has been enhanced to provide network-assisted functional predictions not only for *A. thaliana* but also for 27 nonmodel plant species using orthology-based projections of nonmodel plant genes. This new application will enable gene prioritization for nonmodel plant species, including many food and energy crops.

## CONSTRUCTION

AraNet v2, which is based on the TAIR10 genome build (9), was constructed using machine learning methods that were applied to 19 distinct types of data as summarized in Table 1. The gold standard data set for co-functional gene pairs, which was used for network training, was generated by pairing *A. thaliana* genes that share pathway annotations in the MetaCyc database version 16.0 (10) or Gene Ontology biological process (GO-BP) annotations (11) with IDA (inferred from direct assay), IPI (inferred from protein interaction), ISS (inferred from sequence or structural similarity) or TAS (traceable author statement) evidence codes. We excluded annotations with IGI (inferred from genetic interaction) and IMP (inferred from mutant phenotype) to reduce between-pathway gene pairs, and IEP (inferred from expression pattern) to avoid biased evaluation toward co-expression links. The resultant 19 networks for individual data types were integrated using a log likelihood score scheme and weighted sum method as was used for the previous version of AraNet (3). The integrated AraNet v2 maps 895 000 co-functional links among 22 894 genes (∼83.5% of the known protein coding genes) and includes 3247 more genes than the previous network, AraNet. More details about the network construction are described in the Supplementary Online Methods. Information about the data sources and inference methods used for the co-functional links in AraNet v2 are also summarized in Supplementary Table 1. Edge information from the integrated AraNet v2 as well as all individual networks for the 19 distinct data types is available from the download page at www.inetbio.org/aranet/.

**Table 1.** Summary of the 19 data types that support the co-functional links in AraNet v2

Supporting evidence for co-functional links	Number of genes (% coding genome)	# links
(AT-CC) co-citation of *Arabidopsis thaliana* genes among MEDLINE abstracts or full-text articles from PubMed Central (as of August 2013)	7760 (28.3)	78 000
(AT-CX) co-expression of *A. thaliana* genes across microarray experiments	21 710 (79.2)	500 595
(AT-DC) domain co-occurrence between *A. thaliana* proteins	7634 (28.4)	25 000
(AT-GN) genomic neighborhood of *A. thaliana* orthologs among prokaryotic genomes	2752 (10.0)	51 000
(AT-HT) interactions between *A. thaliana* proteins measured by high-throughput experiments	4216 (15.3)	8000
(AT-LC) interactions between *A. thaliana* proteins from the literature	2709 (9.9)	5168
(AT-PG) phylogenetic profile similarity between *A. thaliana* genes	3613 (13.2)	66 000
(CE-CC) co-citation of *C. elegans* orthologs among full-text articles from PubMed Central	2630 (9.6)	64 214
(CE-CX) co-expression of *C. elegans* orthologs across microarray experiments	3835 (14.0)	63 000
(DM-CX) co-expression of *D. melanogaster* orthologs across microarray experiments	3968 (14.5)	96 000
(DR-CX) co-expression of *D. rerio* orthologs across microarray experiments	2182 (8.0)	46 000
(HS-CX) co-expression of *H. sapiens* orthologs across microarray experiments	4973 (18.1)	120 000
(HS-HT) interactions between *H. sapiens* orthologs measured by high-throughput experiments	3855 (14.1)	46 000
(HS-LC) interactions between *H. sapiens* orthologs from the literature	6148 (22.4)	87 000
(SC-CC) co-citation of *S. cerevisiae* orthologs among MEDLINE abstracts	4124 (15.0)	96 152
(SC-CX) co-expression of *S. cerevisiae* orthologs across microarray experiments	2964 (10.8)	71 000
(SC-GT) similarity of genetic interactions between *S. cerevisiae* orthologs	2179 (7.9)	19 000
(SC-HT) interactions between *S. cerevisiae* orthologs measured by high-throughput experiments	2776 (10.1)	57 000
(SC-LC) interactions between *S. cerevisiae* orthologs from the literature	4361 (15.9)	89 866
(AraNet v2) integrated network	22 894 (83.5)	895 000

## ASSESSMENT AND APPLICATIONS

### AraNet v2 assessment

**Figure 1.**
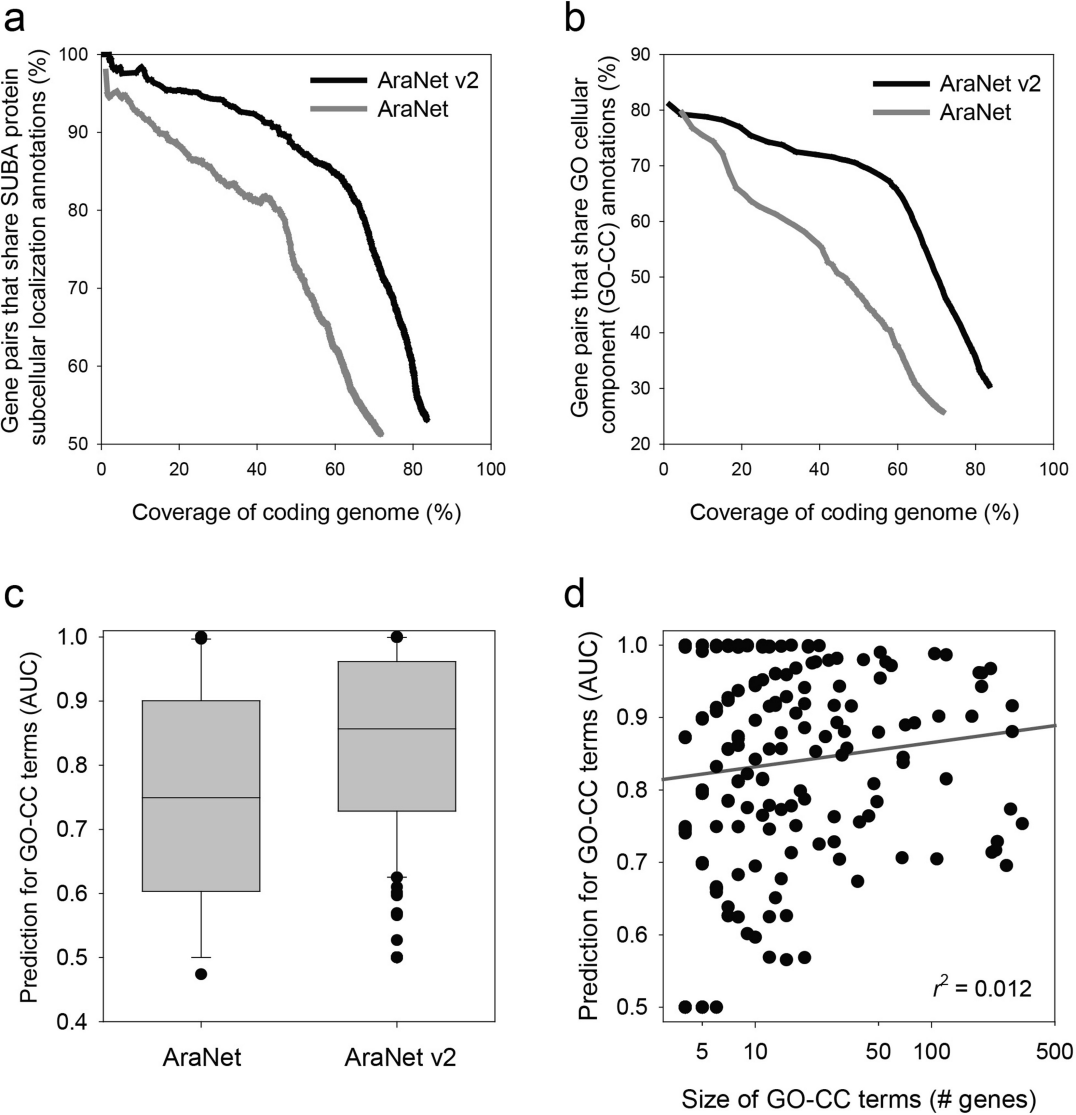
Network assessment using a set of validation gene pairs based on SUBA3 (**a**) and GO-CC (**b**). The accuracy of the co-functional links of each network was calculated as the percentage of true positives for each bin of 1000 gene pairs. The resultant plot shows that AraNet v2 outperforms AraNet for the entire genome coverage range. (**c**) A box-and-whisker plot of network prediction power for 212 GO-CC terms with more than four annotated genes, measured by area under the curve from ROC analysis. AraNet v2 is also superior to the previous network in prediction for GO-CC annotations. (**d**) x-axis and y-axis represent the size of each GO-CC term and measured prediction power for the terms by AUC, respectively. These two variables have no significant correlation (*r^2^* = 0.012), indicating no impact of gene set size on network prediction power.

If the accuracy of the co-functional links in AraNet v2 is equal or higher than AraNet, then we expect that the expansion of the genome coverage in AraNet v2 may lead to enhanced predictions for functions and phenotypes in *A. thaliana*. We used a validation set, which was composed of gene pairs independent from the gold standard gene pairs used for network training, to assess the predictive power of each network. Assuming two genes participating in the same pathway are likely to belong to the same protein complex or to be localized within the same subcellular compartment, we generated two distinct validation sets: (i) gene pairs that share subcellular localization annotations by SUBcellular localization database for *Arabidopsis* proteins (SUBA3) (12), (ii) gene pairs that share GO cellular component (GO-CC) annotations (11). To avoid misleading co-functional relationships due to ambiguous subcellular compartments, we ignored the SUBA3 annotations for ‘cytosol’ and ‘plasma membrane’. For the same reason, we excluded 16 GO-CC terms with more than 350 annotated genes from a total of 420 terms. These procedures resulted in 335 641 gene pairs by SUBA3, of which only 418 pairs overlapped with the gold standard gene pairs that we used for network training (∼0.3%), and 394 076 gene pairs by GO-CC, of which less than 9% overlapped with the gold standard gene pairs. The accuracy of the network links for the given genome coverage was compared between AraNet v2 and AraNet based on the validation set. A substantially higher accuracy over the entire genome coverage range was observed using AraNet v2 for both SUBA3 and GO-CC annotations (Figure 1a and b). From this result, we conclude that AraNet v2 significantly improves both genome coverage and linkage accuracy.

We also measured network prediction power for each GO-CC terms with leave-one-out analysis setting (13), in which a gene for a GO-CC term is prioritized by network connections to all other member genes. Retrieval rate of true positive member genes for each GO-CC terms was measured by receiver operating characteristic (ROC) analysis, which is generally summarized as area under the ROC curve (AUC) score. We observed significantly improved prediction power of AraNet v2 for GO-CC terms compared to the previous AraNet (*P* < 1 × 10^−16^, Wilcoxon signed rank test) (Figure 1c). Next, we examined if the prediction power of AraNet v2 is affected by the number of annotated genes for the GO-CC terms to predict. We observed no significant correlation between the size of GO-CC terms and AUC (Figure 1d), suggesting no significant impact of size of gene set on the observed prediction power of AraNet v2.

There are many updates to data sources and analysis methods, as summarized in Supplementary Table 1 that may have contributed to the improvements observed with AraNet v2. For example, three major updates in data sources were included in AraNet v2: microarray data for co-expression links, high-throughput protein–protein interaction data and co-citation links. Co-functional links based on co-expression were inferred from 64 series comprising a total of 1261 microarray experiments from the Gene Expression Omnibus (GEO) database (4) in AraNet v2. In contrast, AraNet included only 11 sets for 242 experiments from the TAIR database (3). In addition, the large-scale protein–protein interaction data sets for *A. thaliana* that were included in AraNet v2 were not publicly available at the time of the publication of AraNet. We have also integrated co-functional gene links from the co-citation of genes among MEDLINE abstracts or PubMed Central full-text articles in AraNet v2, a method that was not used during the development of AraNet. Improved analysis methods for genome context data may have also contributed to the improved performance of AraNet v2.

### The study of Arabidopsis and 27 nonmodel plants using the AraNet v2 web server

The guilt-by-association principle has proven to be an effective approach for generating novel functional hypotheses based on gene networks (14). For example, the previous AraNet successfully identified novel *A. thaliana* genes for seed pigmentation, root growth and drought stress resistance (3). Due to the substantial improvement in both genome coverage and accuracy, we anticipate more effective predictions using AraNet v2. A web server with network search functions can not only provide a database of co-functional networks but also enable biologists to generate novel hypotheses. We have updated the web server, therefore, to enhance the usability of the improved network database in AraNet v2. The AraNet web server provides two network search options: (i) ‘Find new members of a pathway’ and (ii) ‘Infer functions from network neighbors’ (15). The ‘Find new members of a pathway’ option searches for new candidate genes associated with a pathway or phenotype of interest. Users first submit a query gene set for a pathway or phenotype. The search option then prioritizes other genes for the phenotype by set-wise connectivity scores (i.e. the sum of the log likelihood scores) to the query genes. Networks of query and candidate genes are visualized by Cytoscape Web (16). In contrast, the ‘Infer functions from network neighbors’ option prioritizes functions, as GO-BP terms, for a query gene. The search option prioritizes the GO-BP terms from the most enriched among neighbors of the query gene in AraNet v2. Based on our experience, we recommend that a user considers the top 10 candidate GO-BP terms for the subsequent functional analysis.

**Figure 2.**
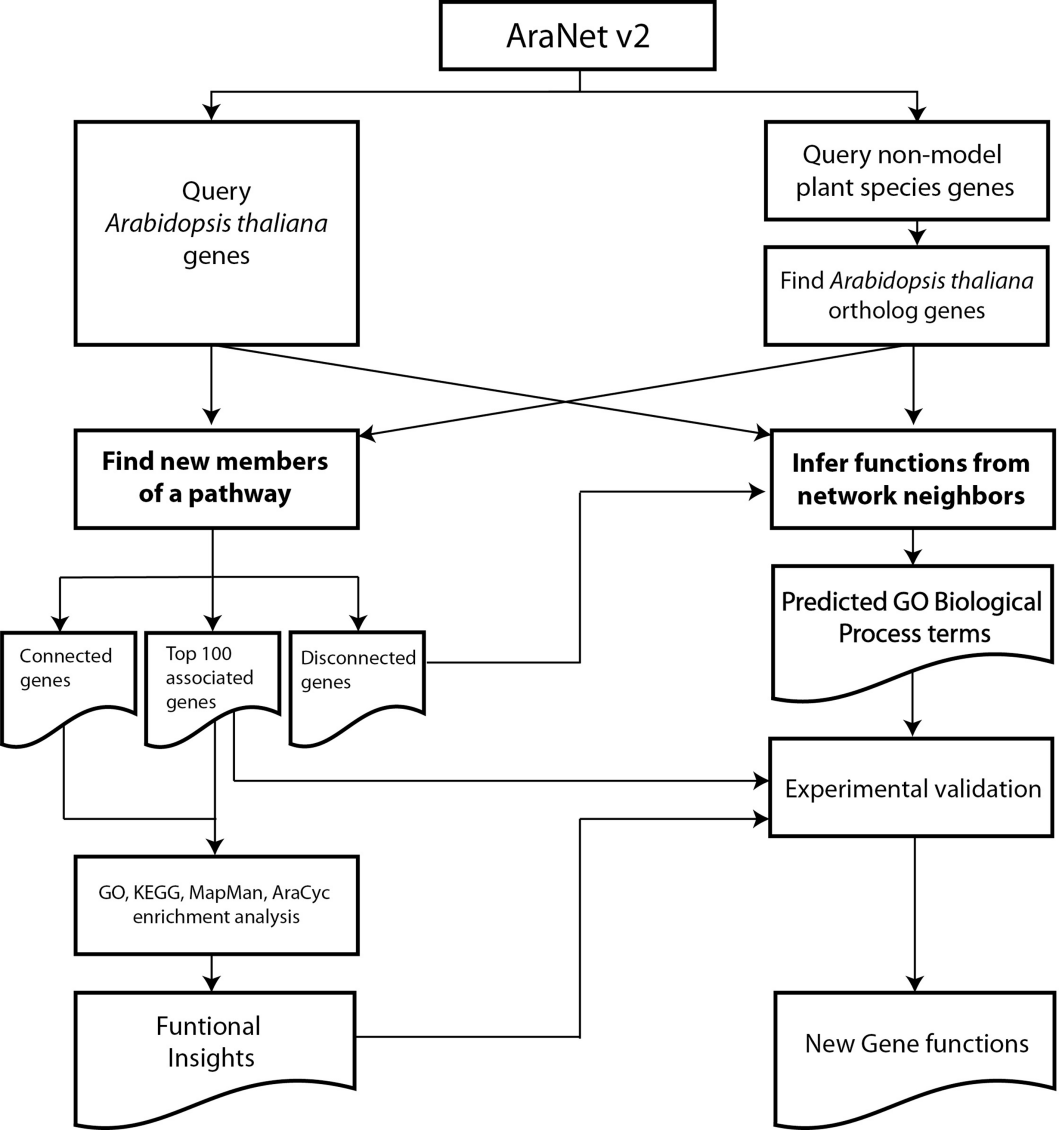
A schematic figure of the two different query processes for the network-assisted hypothesis generation implemented in AraNet v2.

Technical barriers to the investigation of nonmodel plant genomics and genetics have been largely circumvented with the advent of NGS. Plant scientists have started to look beyond the model plant, *Arabidopsis*, to other nonmodel plant species such as food and energy crops. As of June 2014, the genomes of more than 30 plant species have been fully sequenced. Systems-level functional models such as gene networks, however, are not available for most nonmodel plants due to a lack of genome-scale functional profile data such as gene expression microarray, RNA sequencing, and genome-scale protein–protein interaction analysis. Given that there has not yet been a significant accumulation of species-specific functional genomics data, network construction using an orthology-based transfer of network information from a model plant, *A. thaliana*, to nonmodel plants may be an effective approach (17–19). Alternatively, genes from a nonmodel plant can be projected onto AraNet v2 by orthology, which would allow for the efficient implementation of a network-assisted functional study of a large volume of nonmodel plant species based on a single gene network. We therefore renovated the AraNet v2 web server to perform functional predictions not only for *A. thaliana* but also for the 27 nonmodel plant species listed in Supplementary Table 2. Because our published functional network for rice (20) is based on the previous version of AraNet, we have decided to include rice for AraNet v2 server. Users can select one of 28 plant species (*A. thaliana* or one of the 27 nonmodel plants) and submit a set of query genes to the network search page of the web server. For queries involving nonmodel plant species, the server provides *A. thaliana* orthologs of the nonmodel plant genes pre-mapped by the best-bidirectional BLASTp hits. This strict orthology was employed to reduce spurious functional inference from prevalent paralogs in plant species. The prediction results show both the original query plant genes and the *A. thaliana* orthologs. The query process implemented for AraNet v2 is summarized in Figure 2. More detailed descriptions about submission methods and interpretations of query results are available from the tutorial page at www.inetbio.org/aranet/.

### Network-assisted gene prioritization for model and nonmodel plant phenotypes

The prediction power of AraNet v2 for *A. thaliana* genes can be demonstrated using an example in which we identify novel candidate genes for the shade avoidance response. We prioritized genes for the shade avoidance response in *A. thaliana* using the ‘Find new members of a pathway’ option by querying 35 *a priori* genes summarized in Filiault *et al.* (21). The roles of these 35 query genes in the vegetative shade avoidance response have been experimentally confirmed, and closely connected genes in the network are good candidates for the same phenotype. To validate the new candidate genes by network-assisted prioritization, we employed 53 *de novo* genes for shade avoidance identified from a genome-wide association study (GWAS), which were also reported by Filiault *et al.* We found six *de novo* genes (*AT1G09530, AT4G32980, AT5G43700, AT5G57360, AT1G15050, AT5G35840*) among the top 20 network-assisted candidates. This result indicates that the top 20 candidates identified by AraNet-assisted prediction are enriched for confident GWAS candidates at a 147-fold higher than random chance (53 *de novo* genes out of 27 416 *A. thaliana* genes). We also performed the ‘Infer functions from network neighbors’ option for the six validated candidates, and found that all of the candidates have at least one GO-BP term known to be relevant to the shade avoidance response, including terms such as ‘gibberellic acid mediated signaling pathway’, ‘response to auxin’, ‘response to blue light’, ‘response to far red light’, among the top five predictions (Supplementary Table 3). From these results, we conclude that AraNet v2 can effectively prioritize novel candidates for complex phenotypes in *A. thaliana.*

**Figure 3.**
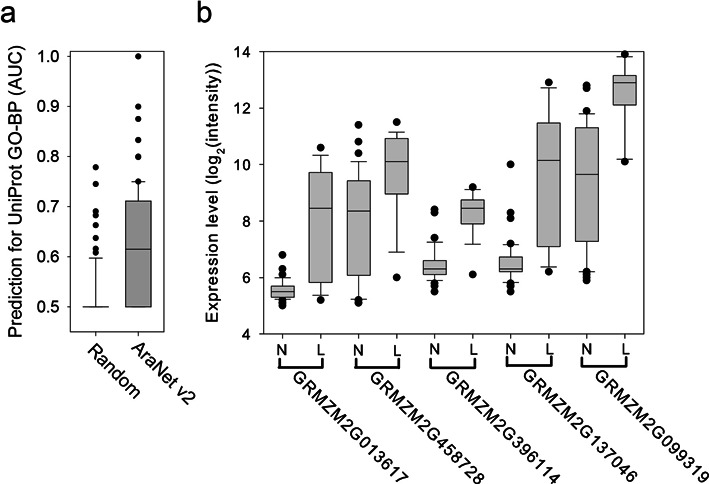
(**a**) A box-and-whisker plot of AraNet v2 prediction power (measure by AUC) for maize UniProt GO-BP annotations and those by a randomized model. (**b**) A box-and-whisker plot that summarizes the expression levels of five genes that were highly ranked among maize leaf initiation candidates across 42 nonleaf tissues (N) and 18 leaf tissues (L). The expression levels were measured by the log base 2 of the intensity value of the hybridized spots. All five genes show significantly elevated expression levels in leaf tissues (by Wilcoxon rank-sum test: GRMZM2G013617, *P* = 1.67 × 10^−5^; GRMZM2G396114, *P* = 5.52 × 10^−4^; GRMZM2G458728, *P* = 8.45 × 10^−8^; GRMZM2G137046, *P* = 8.56 × 10^−7^; GRMZM2G099319, *P* = 2.75 × 10^−7^).

Next, we tested whether AraNet v2 can predict genes for phenotypes in nonmodel plant species. To assess general prediction power of AraNet v2 for nonmodel plants, we performed the ‘Find new members of a pathway’ option for 85 maize GO-BP terms annotated by UniProt-GO Annotation database (22). We found that AraNet v2 achieved AUC > 0.7 for more than 25% of tested maize GO-BP terms (median AUC = 0.61), whereas randomized model showed no prediction power for most of the tested terms (Figure 3a). Particularly, we have searched for novel candidate genes involved in maize leaf initiations by running ‘Find new members of a pathway’ option with 17 maize genes known for the process (23). Among the top candidate genes, we found a known gene for maize leaf adaxial-abaxial patterning, GRMZM2G082264 (rank 16) (24), which is relevant to the regulation of leaf architecture. We also utilized the Maize Gene Expression Atlas (25), which provides maize gene expression data across 60 distinct tissues representing 11 major organs of inbred B73. We hypothesized that genes for leaf initiation are expressed significantly more in leaf tissues than in other tissues. To test this hypothesis, we collected gene expression data for the 60 distinct tissues from the Maize Genome DataBase (MaizeGDB) (26), and compared their distributions of expression levels between leaf and nonleaf tissues. We observed a significantly higher range of expression levels for five high-ranked genes [GRMZM2G013617 (rank 1), GRMZM2G458728 (rank 2), GRMZM2G396114 (rank 3), GRMZM2G137046 (rank 10) and GRMZM2G099319 (rank 12)] (Figure 3b) in 18 leaf tissues compared with 42 nonleaf tissues. Taken together, we conclude that AraNet v2 can also effectively predict candidate genes for nonmodel plant species.

## CONCLUSION

AraNet v2 is a freely accessible database of co-functional links between *A. thaliana* genes. This updated version of the network shows significant improvement in network quality and functional prediction power. The results of our network assessment suggest that continued efforts in generating large-scale omics data and improving data analysis algorithms will further increase the power of network biology. In addition, through the use of renovated web server applications, a single network for AraNet v2 can facilitate predictive genetics not only for a model plant such as *A. thaliana* but also for many nonmodel plant species. Genome-wide genetics studies for nonmodel plants are more accessible due to the power of high-throughput DNA sequencing technology, and functional genomics data are generally complementary to information from the genetics studies. Network-assisted functional inference for nonmodel plants using AraNet v2 may therefore be able to complement genome-wide genetics studies, which will contribute significantly to the genetic dissection of complex phenotypes of food and energy crops in the future.
